# Pre-treatment calprotectin (MRP8/14) provides no added value to testing CRP alone in terms of predicting response to TNF inhibitors in rheumatoid arthritis in a post hoc analysis

**DOI:** 10.1136/ard-2022-222519

**Published:** 2023-02-21

**Authors:** Samantha Louise Smith, Sheree Alexander, Nisha Nair, Sebastien Viatte, Stephen Eyre, Kimme L Hyrich, Ann W Morgan, Anthony G Wilson, John D Isaacs, Darren Plant, Anne Barton

**Affiliations:** 1 Centre for Genetics and Genomics Versus Arthritis, Centre for Musculoskeletal Research, Manchester Academic Health Science Centre, The University of Manchester, Manchester, UK; 2 NIHR Manchester Biomedical Research Centre, Manchester University NHS Foundation Trust, Manchester Academic Health Science Centre, Manchester, UK; 3 Lydia Becker Institute of Immunology and Inflammation, Division of Musculoskeletal and Dermatological Sciences, School of Biological Sciences, Faculty of Biology, Medicine and Health, The University of Manchester, Manchester, UK; 4 Centre for Epidemiology Versus Arthritis, Centre for Musculoskeletal Research, Manchester Academic Health Sciences Centre, The University of Manchester, Manchester, UK; 5 School of Medicine, University of Leeds and NIHR Leeds Musculoskeletal Biomedical Research Centre, Leeds Teaching Hospitals NHS Trust, Leeds, UK; 6 UCD School of Medicine and Medical Science, Conway Institute, University College Dublin, Dublin, Ireland; 7 Musculoskeletal Research Group, Translational and Clinical Research Institute, Newcastle University and NIHR Newcastle Biomedical Research Centre, Newcastle upon Tyne Hospitals NHS Foundation Trust, Newcastle upon Tyne, UK

**Keywords:** Arthritis, Rheumatoid, Biological Therapy, Tumor Necrosis Factor Inhibitors

## Abstract

**Objectives:**

The inflammatory protein calprotectin (MRP8/14) has been identified as a promising biomarker of treatment response in rheumatoid arthritis (RA). Our aim was to test MRP8/14 as a biomarker of response to tumour necrosis factor (TNF)-inhibitors in the largest RA cohort to date and to compare with C-reactive protein (CRP).

**Methods:**

Serum MRP8/14 was measured in 470 patients with RA about to commence treatment with adalimumab (n=196) or etanercept (n=274). Additionally, MRP8/14 was measured in the 3-month sera of 179 adalimumab-treated patients. Response was determined using European League against Rheumatism (EULAR) response criteria calculated using the traditional 4-component (4C) DAS28-CRP and alternate validated versions using 3-component (3C) and 2-component (2C), clinical disease activity index (CDAI) improvement criteria and change in individual outcome measures. Logistic/linear regression models were fitted for response outcome.

**Results:**

In the 3C and 2C models, patients with RA were 1.92 (CI: 1.04 to 3.54) and 2.03 (CI: 1.09 to 3.78) times more likely to be classified as EULAR responders if they had high (75th quartile) pre-treatment levels of MRP8/14 compared with low (25th quartile). No significant associations were observed for the 4C model. When only using CRP as a predictor, in the 3C and 2C analyses, patients above the 75th quartile were 3.79 (CI: 1.81 to 7.93) and 3.58 (CI: 1.74 to 7.35) times more likely to be EULAR responders and addition of MRP8/14 did not significantly improve model fit (p values=0.62 and 0.80, respectively). No significant associations were observed in the 4C analysis. Exclusion of CRP from the outcome measure (CDAI) did not result in any significant associations with MRP8/14 (OR 1.00 (CI: 0.99 to 1.01), suggesting that the associations were due to the correlation with CRP and that there is no additional utility of MRP8/14 beyond use of CRP in patients with RA starting TNFi therapy.

**Conclusion:**

Beyond correlation with CRP, we found no evidence to suggest that MRP8/14 explains additional variability in response to TNFi in patients with RA over and above CRP alone.

**WHAT IS ALREADY KNOWN ON THIS TOPIC**

- MRP8/14 has gained interest as a biomarker predictive of successful biological disease-modifying anti-rheumatic drug therapy in rheumatoid arthritis (RA) in multiple studies. However, some studies have also reported no association with response.

**WHAT THIS STUDY ADDS**

- This is the largest replication study conducted to date in RA, where we have shown no association between pre-treatment MRP8/14 and response to tumour necrosis factor (TNF) inhibitors (TNFis) beyond the use of C-reactive protein alone.

**HOW THIS STUDY MIGHT AFFECT RESEARCH, PRACTICE OR POLICY**

- Although MRP8/14 was not found as predictive of TNFi response in patients with RA in this study, MRP8/14 is thought to be a sensitive marker of synovitis. As such, further critical investigation is needed to determine its use in composite disease activity scores, such as DAS28, compared with the use of traditional acute phase reactants.

## Introduction

Calprotectin (also known as MRP8/14 or S100A8/A9) is a protein complex consisting of two monomers found in serum/plasma and faeces. Released locally at sites of inflammation by granulocytes/monocytes, it is an established biomarker for monitoring disease activity in inflammatory bowel disease (IBD) and has been reported to predict response in patients with IBD treated with biological therapies.^1^ As such, MRP8/14 has gained interest in other inflammatory disorders, including rheumatoid arthritis (RA) and juvenile idiopathic arthritis (JIA), where it has been found to be upregulated in inflamed synovial fluid and blood.^2–8^

Biological disease-modifying anti-rheumatic drugs (bDMARDs) have significantly improved the management of RA. However, response is not universal, with unsatisfactory control of disease symptoms experienced by 30%–40% of treated patients.^9^ Identification of biomarkers that predict response would allow more directed and effective prescribing. However, to date no reliable biomarkers have been established.

Due to the strong correlation between MRP8/14 and measurements of disease activity, including ultrasonography (US),^3 10–15^ MRP8/14 may represent a good candidate biomarker for monitoring response to treatment. Indeed, several studies have reported that MRP8/14, measured before treatment initiation, predicts response to both bDMARDs and conventional synthetic DMARDs (csDMARDs) in patients with RA and JIA.^11 12 16–22^ Specifically in RA, MRP8/14 has been reported to correlate well with response to tumour necrosis factor (TNF) inhibitors (TNFis) and rituximab—with good responders presenting with higher pre-treatment MRP8/14.^16^ Several studies have also shown that MRP8/14 levels decrease following successful treatment.^11 12 16 19 21 23 24^

However, others have also reported conflicting results with no correlation with treatment response.^15 23 25 26^ Before this marker can be adopted into a clinical setting, it is important to validate in larger independent cohorts. As such, the aim of this study was to determine the relationship between pre-treatment serum MRP8/14 and response to TNFis in the largest RA cohort to date and, in a subset of samples, investigate changes in serum levels following treatment initiation.

## Materials and methods

### Patient selection

Patients with RA were selected from the Biologics in Rheumatoid Arthritis Genetics and Genomics Study Syndicate (BRAGGSS), which recruits patients who are about to commence treatment with a bDMARD from more than 50 sites across the UK, previously described in detail.^27^ Patients provide blood samples for biomarker measurements and psychological/clinical information at pre-treatment and after 3, 6 and 12 months on therapy. As such, disease activity scores, such as the disease activity score in 28 joints (DAS28) and the clinical disease activity index (CDAI) are calculated prior to and following treatment.^28 29^

Inclusion criteria for this study were as follows: participants were (1) of European descent, (2) aged 18 years or over, (3) fulfilled the 1987 ACR criteria for RA, (4) about to commence treatment with either etanercept (ETN) or adalimumab (ADA), (5) had pre-treatment and follow-up disease activity scores, (6) had a pre-treatment serum sample and (7) for subsets of participants a paired 4-week or 3-month serum sample. Informed consent was obtained from all subjects.

Serum samples from 19 healthy individuals were also selected from the National Repository for Healthy Volunteers (NRHV) study within the Versus Arthritis Centre for Genetics and Genomics at the University of Manchester. Healthy controls were age, gender and ethnically matched to the RA samples.

### Definition of treatment response

Response to treatment was assessed following 3 months on treatment using (1) the European League against Rheumatism (EULAR) response criteria,^30^ (2) CDAI response criteria^31^ and (3) continuous measures (such as change in DAS28 or change in swollen joint count (SJC)). DAS28-C reactive protein (CRP) was calculated using four variables (the number of tender and swollen joints, CRP and patient’s global assessment of disease activity on a visual analogue scale (VAS)),^28^ three variables (the number of tender and swollen joints and CRP)^32^ and two variables (number of swollen joints and CRP)^33^ and were used to define EULAR response categories. A good response was defined as having a follow-up DAS28-CRP of ≤3.2 and a decrease from pre-treatment of >1.2. A non-response was defined as having a DAS28-CRP score that decreased <0.6 from the pre-treatment or decreased between 0.6 and 1.2 but having a follow-up DAS28-CRP score of >5.1. Moderate response was classified when responses fell between these two extremes. For some analyses, response groups were merged (eg, good and moderate responders vs non-response). CDAI was calculated as the sum of four variables: tender and swollen joint counts, patient global assessment of disease activity and evaluator global assessment of disease activity. Using CDAI, a non-response was defined as <50% improvement from pre-treatment, a mild response of ≥50% and <70% improvement, a moderate response of ≥70% and <85% improvement and a major response as >85% improvement.^31^ For the continuous analyses, response was assessed as the change between pre-treatment and 3 months.

### Sample collection

Samples for serology were collected into plain blood tubes and were shipped from national recruiting centres at ambient temperature to the Versus Arthritis Centre for Genetics and Genomic at the University of Manchester. On receipt, samples were centrifuged at 3000 rpm for 10 min and the serum aliquoted. Samples were logged onto the laboratory information management system (LIMS) and stored at −80°C until required.

### Measurement of MRP8/14

Serum MRP8/14 was determined using the CE-marked IVD BÜHLMANN *MRP8/14* ELISA Kit (Alpha Laboratories, UK) according to the manufacturer’s instructions. All samples and standards were assayed in duplicate. The detection range for the assay was 0.4 µg/mL to 24 µg/mL; if a concentration outside this range was recorded, the sample was diluted and the assay repeated. Similarly, if duplicate samples differed by >20%, the assay was repeated. Where a paired pre-treatment and 4-week sample were available, MRP8/14 on the paired samples was determined using the LEGEND MAX Human MRP8/14 (Calprotectin) ELISA Kit (BioLegend, San Diego, USA). Prior to using the BioLegend LEGEND MAX ELISA, pre-treatment RA samples (n=13) were assayed on both systems, where a strong correlation (R^2^=0.93) was observed. Absorbance was measured using the SpectraMax Plus^384^ Absorbance Microplate Reader (Molecular Devices, California, USA) with 450 nm as the primary wavelength.

### Statistical analysis

All analyses were conducted in STATA/SE V.14.0^34^ or Prism V.8.1.2 (GraphPad Software). Significance between treatment groups and baseline characteristics were compared using χ^2^ test, two independent samples t-test or Mann-Whitney tests. Spearman rank correlations were assessed to determine the relationship between pre-treatment MRP8/14 and clinical parameters. MRP8/14 differences between the EULAR/CDAI response groups/baseline and follow-up time points were compared using Mann-Whitney test statistics. Logistic regression was used to assess the association between pre-treatment MRP8/14 (or CRP) and EULAR non-responders and grouped responders. Ordered logistic regression was used to assess the association between pre-treatment MRP8/14 (or CRP) and CDAI response groups. Linear regression was used to assess the relationship between pre-treatment MRP8/14 and DAS28-CRP or changes in individual outcome measures. A p value of <0.05 was considered statistically significant for all analyses.

### Patient involvement

A biological marker which can predict response to treatment is a priority of our Research User Network (RUN) which was established by the Centre for Musculoskeletal Research in 2009. RUN is a patient–partner initiative supported by the University of Manchester, the NIHR Biomedical Research Centre and Versus Arthritis. The user group is comprised of people with an active interest in musculoskeletal health; either patients who live with a musculoskeletal condition (such as inflammatory arthritis, lupus or fibromyalgia) or people who care for someone with these conditions. Consultation with our RUN has shaped the research we do in the centre.

## Results

In total, 470 patients were treated with a TNFi and had pre-treatment serum MRP8/14. Of these, 196 were treated with ADA and 274 with ETN. In addition, 179 patients receiving ADA also had a paired 3-month serum MRP8/14 measurement. Baseline characteristics are summarised in table 1. Age and sex are representative of a typical RA cohort. Between the ADA and ETN treatment groups, a higher proportion of patients within the ADA treatment group were receiving concomitant csDMARDs.

**Table 1:** Baseline characteristics of patients enrolled in the study

Characteristics	TNFi (n=470)	ADA only (n=196)	ETN only (n=274)
Sex (F), n (%)	363 (77.2)	151 (77.0)	212 (77.4)
Age at baseline (years), mean (SD), n	56.7 (11.9), 469	56.5 (11.9), 195	56.9 (12), 274
BMI, median (IQR), n	27.6 (23.7–32.5), 396	27.5 (23.5–32.6), 164	27.7 (23.9–23.3), 232
Concurrent csDMARDs, n (%)	393 (83.8)	172 (88.2)	221 (80.7)
Baseline DAS28, mean (SD), n	5.8 (0.9), 434	5.7 (0.9), 178	5.8 (0.9), 256
Tender joint count, median (IQR)	15 (10–21)	15 (11–21)	14 (10–21)
Swollen joint count, median (IQR)	8 (5-12)	8 (2–12.5)	9 (5-12)
CRP (mg/L), median (IQR)	9.2 (3.7–25.8)	8.3 (2.8–25.7)	9.6 (4.2–26.7)
HAQ, mean (SD), n	1.7 (0.6), 416	1.7 (0.6), 175	1.6 (0.6), 241

ADA, adalimumab; CRP, C reactive protein; csDMARDs, conventional synthetic disease-modifying anti-rheumatic drugs; DAS28, disease activity score in 28-joints calculated using 4-components; ETN, etanercept; F, female; HAQ, Health Assessment Questionnaire; IQR, interquartile range; SJC, swollen joint count; TJC, tender joint count; TNFi, tumour necrosis factor inhibitor.

Univariable ordered logistic regression analysis was used to assess associations between clinical parameters prior to starting treatment and EULAR response groups defined using the 4-component DAS28-CRP scores (4C-DAS28-CRP). Of note, 4C-DAS28-CRP EULAR response groups were only available on 434/470 patients (online supplemental table 1). HAQ score was significantly associated with response in the cohort as a whole (hence referred to as TNFi) and after stratifying by drug type (ADA and ETN), with good responders presenting with lower pre-treatment disability scores compared with moderate and non-responders, respectively (TNFi: 1.5 vs 1.8 and 1.7 (OR 0.53 (95% CI: 0.38 to 0.73), p<0.001); ADA: 1.5 vs 1.8 and 1.8 (OR 0.47 (95% CI: 0.28 to 0.81), p value=0.007); ETN: 1.4 vs 1.7 and 1.7 (OR 0.54 (95% CI: 0.36 to 0.80), p value=0.003). BMI was also associated with EULAR response but only in the ADA treatment arm, with higher BMIs associated with non-response (32.3 for non-responders vs 28.2 and 27.0 for moderate and good responders, respectively; p value=0.002). Additionally, concurrent csDMARD use was associated with EULAR response but again only in the ADA treatment arm; those not on concurrent therapy were 3.79 times at greater risk (95% CI: 1.60 to 8.96) of being a non-responder compared with moderate and good responders (p value=0.002).

10.1136/ard-2022-222519.supp1 Supplementary data

### Calprotectin is associated with baseline markers of disease activity

**Figure 1:**
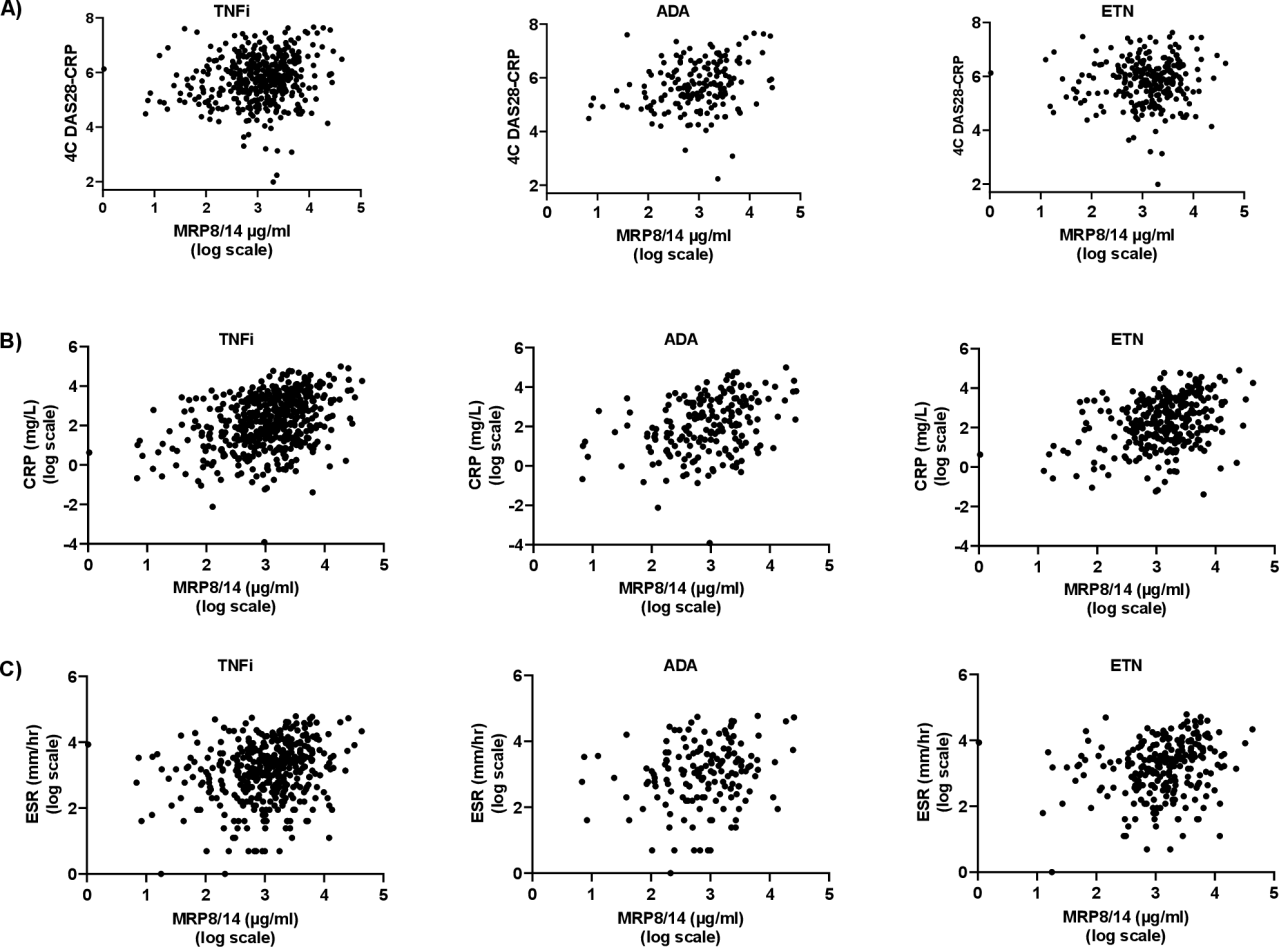
Scatterplots showing the correlation between pre-treatment serum levels of MRP8/14 and parameters of baseline disease activity: (A) DAS28-CRP calculated using 4-components, (B) CRP and (C) ESR. ADA, adalimumab; CRP, C reactive protein; DAS28, disease activity scores in 28-joints; ESR, erythrocyte sedimentation rate; ETN, etanercept; TNFi, tumour necrosis factor inhibitor.

In line with previous results, associations between pre-treatment MRP8/14 and measurements of pre-treatment disease activity were observed. Levels of MRP8/14 were modestly correlated with 4C-DAS28-CRP (Spearman’s rho=0.15, p value=0.0017), CRP (Spearman’s rho=0.37, p<0.0001) and erythrocyte sedimentation rat (ESR) (Spearman’s rho=0.24, p<0.0001) for TNFi and after stratifying by drug type (figure 1 and online supplemental figure 1). No correlations between pre-treatment MRP8/14 and swollen nor tender joint count were observed in this study (online supplemental figure 2).

### Higher proportion of EULAR responders have high pre-treatment MRP8/14

**Figure 2:**
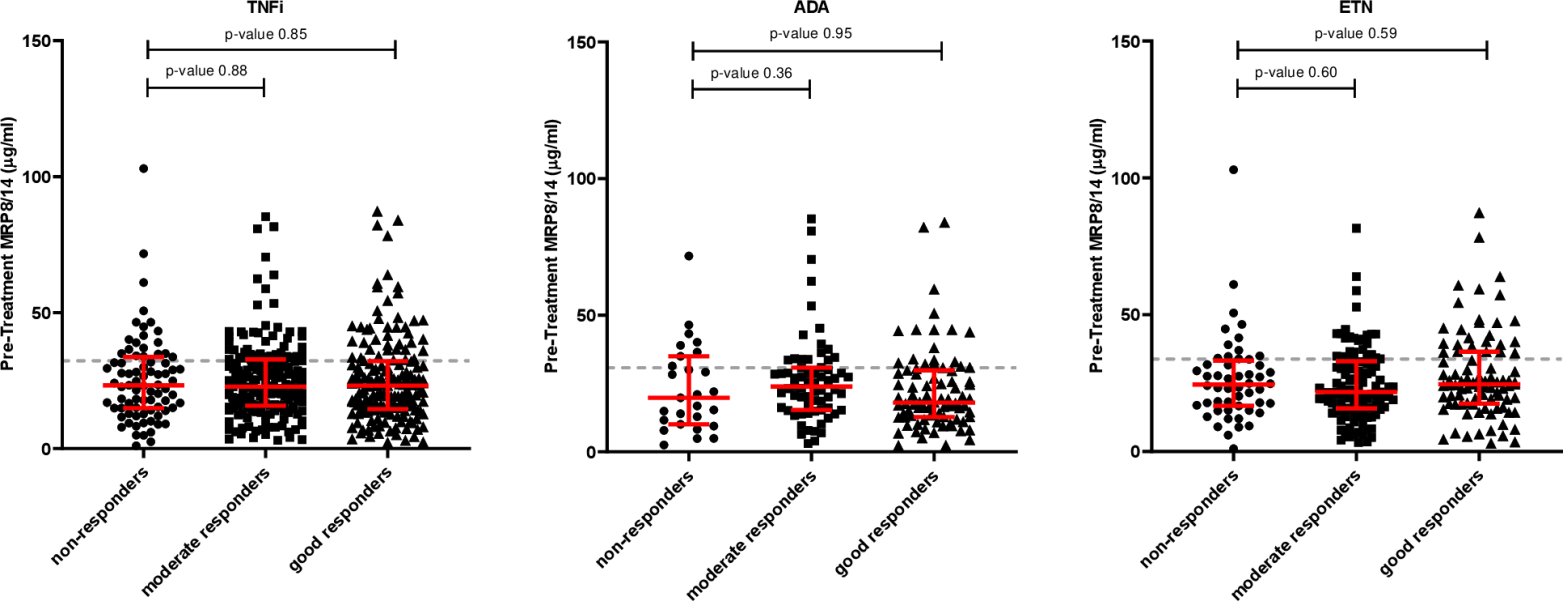
Pre-treatment serum MRP8/14 against EULAR non-responders, moderate responders and good responders, classified at 3-month follow-up to TNF inhibitors as a whole (left plot), adalimumab (ADA) only (middle plot) and etanercept (ETN) only (right plot). EULAR responses calculated using 4-component DAS28. Each dot represents a patient. The data is represented as median and interquartile range. Significance was determined using Mann-Whitney non-parametric test. The grey dotted line represent the 75th quartile. EULAR, European League against Rheumatism; TNFi, tumour necrosis factor inhibitor.

Next, we correlated pre-treatment MRP8/14 with EULAR response categories. MRP8/14 was significantly higher in patients with RA compared with healthy controls (median MRP8/14 in healthy samples was 3.17 µg/mL (2.37–3.83)) (online supplemental figure 3), however, we observed no associations between MRP8/14 and the EULAR response categories using the TNFi cohort as a whole or after stratification by drug type (figure 2).

As MRP8/14 has been shown to correlate strongly with markers of synovitis,^11 13^ we decided to re-calculate EULAR responses using an alternate validated version of the DAS28 using 3-components (which excludes the subjective patient global VAS).^32^ Additionally, we assessed response categories calculated using a recently proposed 2-component DAS28 score (which only includes the SJC and CRP).^33^ Response categories could be calculated for all 470 patients using the 3-component and 2-component scores (hence forth referred to as 3C and 2C) (online supplemental table 1). Strong correlations were observed between all pre-treatment scores and changes due to treatment (online supplemental figure 4).

**Figure 3:**
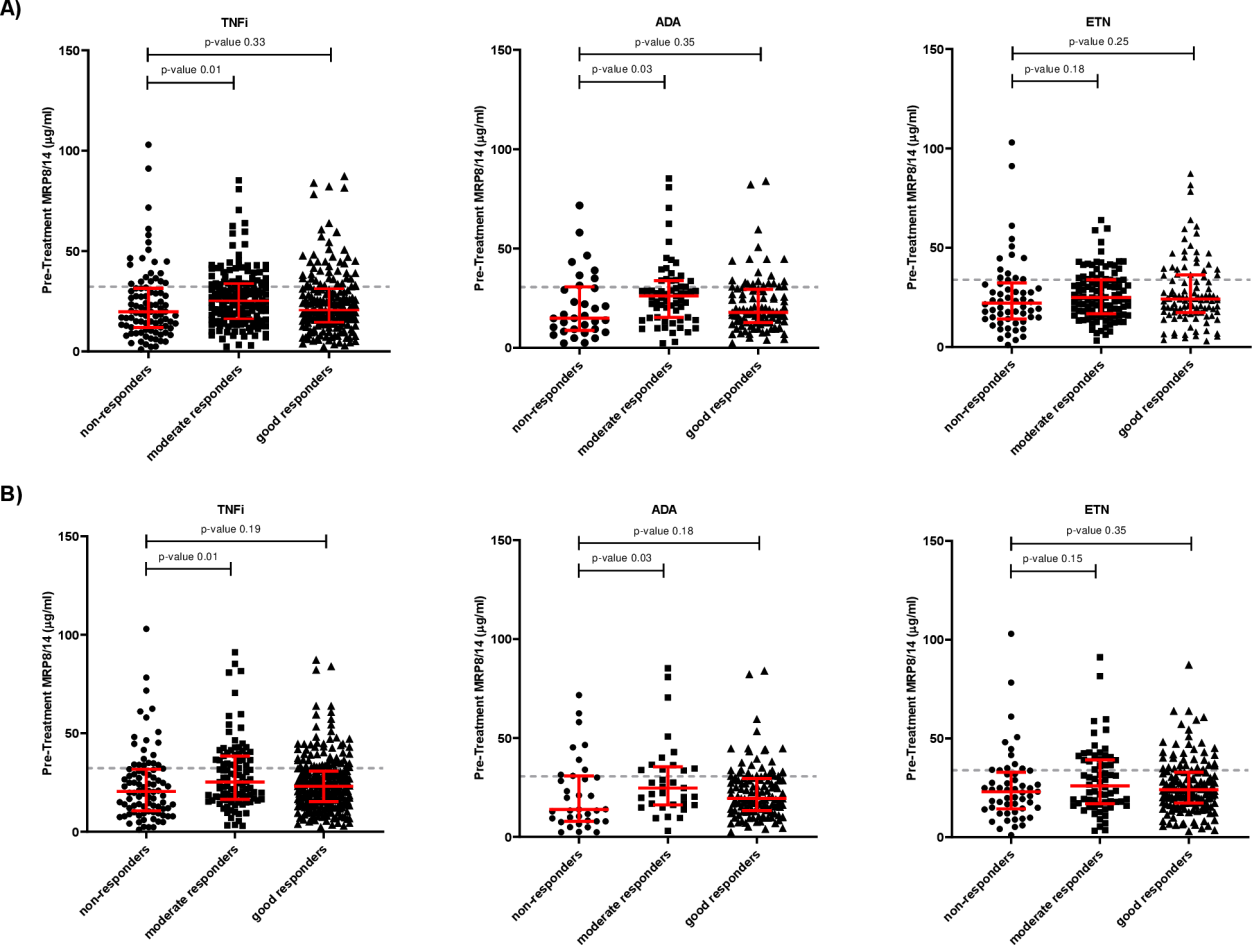
Pre-treatment serum MRP8/14 against response categories: non-responders, moderate responders and good responders, classified at 3-month follow-up to TNF inhibitors as a whole (left plot), adalimumab (ADA) only (middle plot) and etanercept (ETN) only (right plot). (A) EULAR responses calculated using 3-component DAS28. (B) Response categories calculated using 2-component DAS28. Each dot represents a patient. The data is represented as median and interquartile range. Significance was determined using Mann-Whitney non-parametric test. The grey dotted line represent the 75th quartile. EULAR, European League against Rheumatism; TNFi, tumour necrosis factor inhibitor.

Following re-analysis, we found that pre-treatment MRP8/14 in patients receiving TNFi were significantly higher in moderate responders compared with non-responders but not between good responders and non-responders. This was consistent for responses calculated using both 3C and 2C (figure 3). In the 3C analysis, moderate responders displayed a median MRP8/14 concentration of 25.3 µg/mL (16.4–33.8), good responders 20.6 µg/mL (14.6–31.2) and non-responders 19.8 µg/mL (12.0–31), while in the 2C analysis, moderate responders displayed a median MRP8/14 concentration of 25.2 µg/mL (16.5–38.5), good responders 23.1 µg/mL (15.4–30.8) and non-responders 20.5 µg/mL (11.0–31.6). Similarly, when stratified by drug type, MRP8/14 was generally higher in future ADA responders compared with non-responders, but this was only significant for non-responders vs moderate responders (figure 3 middle plots). No associations between pre-treatment MRP8/14 and response to ETN were observed (figure 3 right-hand plots). Due to the correlation between MRP8/14 and CRP, we hypothesised that a proportion of moderate responders may display higher pre-treatment CRP. However, this was also observed in the ETN treatment arm, where no association with MRP8/14 was observed (online supplemental figure 5).

As in previous studies,^18^ regardless of how DAS28 scores were calculated, there was an increased frequency of responders (moderate and good) with higher MRP8/14 concentrations prior to treatment compared with non-responders. Setting a high concentration threshold at the 75th quartile per analyses (grey dotted lines figures 2 and 3), over 80% of cases were found to be responders to treatment. For the 4C analysis, 81.8% of patients were responders to TNFi and 81.4% and 81.3% were responders in the ADA and ETN treatment subgroups, respectively. Similarly, in the 3C analysis, 82.5% of patients were TNFi responders and 83.7% and 80.9% were ADA and ETN responders. In the 2C analysis, 83.3% were responders to TNFi and 81.6% and 83.8% were responders to ADA and ETN.

### Utility of MRP8/14 in addition to CRP

As pre-treatment CRP and MRP8/14 are correlated (figure 1B), we next assessed which measure best explained variability in treatment response. In a univariate analysis, pre-treatment MRP8/14 was not associated with response when non-responders were compared with moderate/good responders, but pre-treatment CRP was modestly associated with response; this was only significant for the 3C and 2C analyses (3C: OR 1.01 (CI: 1.00 to 1.03), p value=0.034 and 2C: OR 1.01 (CI: 1.00 to 1.03), p value=0.044) (online supplemental table 2). No association between CRP and response was observed in the 4C analysis (p value=0.475). This appeared to be explained by the inclusion of the VAS in the 4C scores, as opposed to a loss of power, as when the 3C and 2C analyses were limited to the 434 cases in which 4C responses were calculable, the associations were qualitatively unchanged (online supplemental table 3).

When dichotomised into low (25th quartile) and high concentrations (75th quartile), for the 4C analysis, we observed no significant associations. However, in the 3C analysis, patients with higher concentrations of MRP8/14 were 1.92 (1.04 to 3.54) times more likely to respond to treatment with TNFi (p value=0.037) compared with low concentrations. This remained significant for the 2C analysis, where patients were 2.03 (1.09 to 3.78) more likely to respond to treatment (p value=0.025). Of note, although the 4C analysis was not statistically significant, the direction of effect was the same (OR 1.22 (0.62 to 2.38)) (table 2).

**Table 2:** Comparison of different logistic regression models for response to TNFi, comparing grouped responders (moderate and good) and non-responders at 3-month follow-up, dichotomised based using high (75th) and low (25th) concentrations of MRP8/14 or CRP

	Responses categories calculated using:											
	4-component DAS28				3-component DAS28				2-component DAS28			
Model	Cases	OR (95% Cl)	Model fit (pseudo R^2^)	P value	Cases	OR (95% Cl)	Model fit (pseudo R^2^)	P value	Cases	OR (95% Cl)	Model fit (pseudo R^2^)	P value
Low vs high MRP8/14 (µg/mL)	218	1.22 (0.62 to 2.38)	0.0015	0.564	241	1.92 (1.04 to 3.54)	0.017	0.037	241	2.03 (1.09 to 3.78)	0.020	0.025
Low vs high MRP8/14 adjusted for pre-treatment CRP	218	1.10 (0.53 to 2.30)	0.0033	0.795	241	1.53 (0.78 to 2.98)	0.026	0.21	241	1.60 (0.82 to 3.15)	0.030	0.171
Low vs high CRP only (mg/L)	215	1.64 (0.78 to 3.4)	0.0092	0.188	234	3.79 (1.81 to 7.93)	0.062	<0.001	234	3.58 (1.74 to 7.35)	0.058	<0.001
Low vs high CRP adjusted for pre-treatment MRP8/14	215	1.79 (0.79 to 4.06)	0.011	0.163	234	4.11 (1.83 to 9.25)	0.063	0.001	234	3.73 (1.70 to 8.19)	0.059	0.001

Response categorises were calculated using 4 components, 3 components or 2 components.

CRP, C reactive protein; DAS28, disease activity scores in 28-joints; TNFi, tumour necrosis factor inhibitor.

A similar trend was observed for dichotomised pre-treatment CRP. No significant associations were observed with the 4C analysis (p=0.188). However, in the 3C and 2C analyses, patients with higher pre-treatment CRP levels were 3.79 or 3.58 times more likely to respond to TNFi (p<0.001) compared with patients with low levels (table 2); again, the direction of effect in the 4C analysis was the same, although not statistically significant (OR 1.64 (0.78 to 3.4)) (table 2). As previously stated, this was determined to be due to the inclusion of the VAS in the 4C-DAS28-CRP calculation as when the 3C and 2C sample sizes were limited to those with 4C-DAS28-CRP response data, the associations remained qualitatively the same (online supplemental table 4).

When the MRP8/14 models were adjusted for pre-treatment CRP, no associations remained significant. However, when the CRP models were adjusted for pre-treatment MRP8/14 the associations for the 2C and 3C analyses remained significant (p values=0.001) (table 2), suggesting that CRP is a better predictor of response category. Using a likelihood ratio test to compare the CRP only and CRP+MRP8/14 models, no statistically significant improvements in model fit were observed (p values=0.62 and 0.80 for the 3C and 2C models, respectively).

Of note, due to the way in which serum samples were collected, where available, as a sensitivity analysis, we also adjusted our models to account for delays in sample processing (the number of days between blood sampling and centrifugation). We found that compared with same day processing, samples processed after 2 days displayed significantly increased levels of MRP8/14 (online supplemental figure 6). Of note, the majority of our samples were processed within 2 days. However, more importantly, we found that time to processing was not a confounder within our analyses and did not materially alter the findings (online supplemental table 5).

The analyses were also repeated following stratification by drug, which did not qualitatively change these results (online supplemental tables 6-8).

Overall, these data suggest that MRP8/14 does not explain any additional variability in response beyond CRP when using the 2C and 3C models and that inclusion of VAS in 4C models attenuates correlations with biological markers of inflammation.

### Removing the effect of CRP from the outcome: CDAI

As we were comparing the utility of MRP8/14 to that of CRP, we wanted to test an additional outcome that was independent of CRP measurements. We therefore repeated the analyses using the CDAI. We found a strong correlation between pre-treatment CDAI and pre-treatment DAS28-CRP scores (rho=0.85, 0.82 and 0.56 for 4C, 3C and 2C, respectively), as well as between change in CDAI and change in DAS28-CRP after 3 months on treatment (rho=0.90, 0.86 and 0.71 for 4C, 3C and 2C, respectively) (online supplemental figure 7).

**Figure 4:**
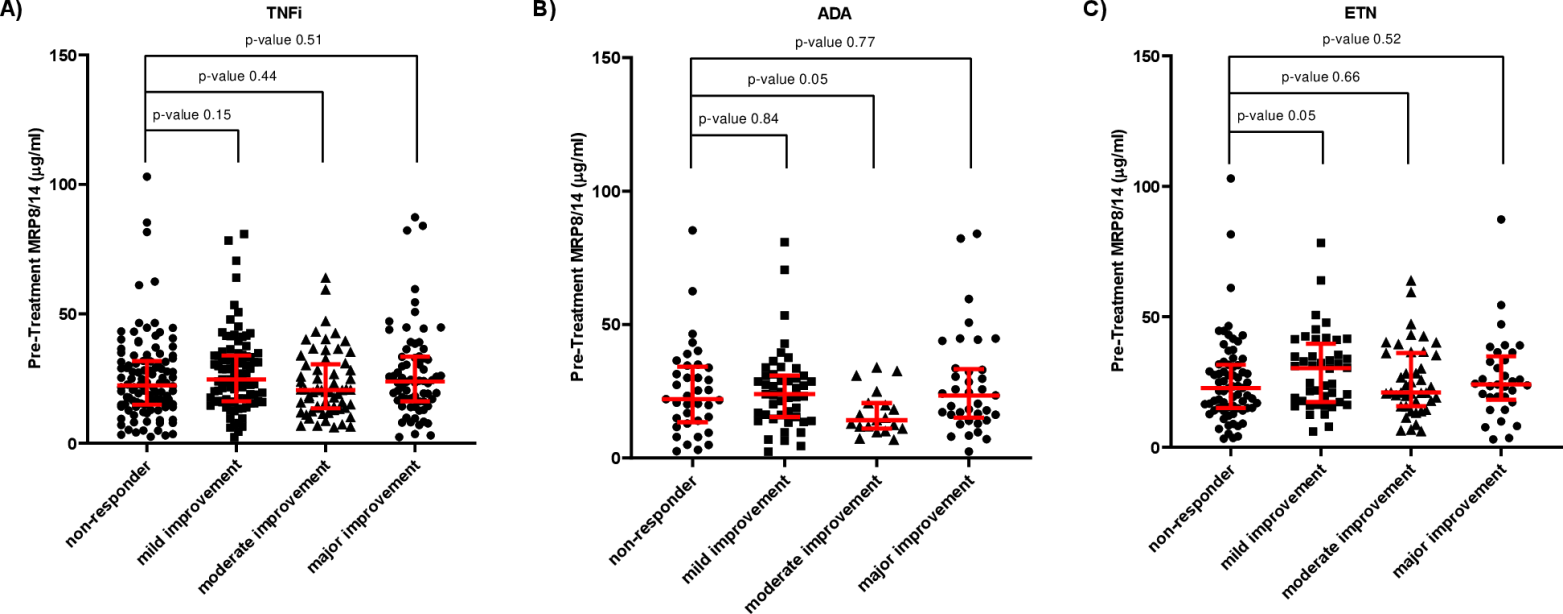
Pre-treatment serum MRP8/14 against CDAI response groups, classified at 3-month follow-up to (A) TNF inhibitors as a whole cohort (TNFi), (B) adalimumab (ADA) only and (C) etanercept (ETN) only. Each dot represents a patient. The data is represented as median and interquartile range. Significance was determined using Mann-Whitney non-parametric test. CDAI, clinical disease activity index; TNFi, tumour necrosis factor inhibitor.

Using pre-treatment CDAI criteria, the majority of patients were classified as having high disease activity (93.7%); the remainder displaying moderate (5.6%) or low disease activity (0.8%). Using the CDAI improvement thresholds to classify response^31^ in 190 ETN and 140 ADA-treated patients, 32.7% of patients were classified as non-responders to TNFi, 27.6% as mild improvers, 18.5% as moderate improvers and 21.2% as major improvers. Pre-treatment MRP8/14 showed no statistically significant association with response (figure 4). Neither did we observe a trend between high MRP8/14 concentrations and CDAI responders, suggesting that the previous associations found with EULAR 3C and 2C responses were driven by CRP.

We used ordered univariable logistic regression to further assess the association between pre-treatment MRP8/14 and CDAI response groups (table 3). No association between MRP8/14 and CDAI response was observed (with and without pre-treatment CRP as an additional predictor variable—likelihood ratio p value=0.51), nor, was any association found after stratifying by drug type (online supplemental table 9).

**Table 3:** Results from ordered logistic regression models to determine the association between pre-treatment MRP8/14 and CDAI responses to TNFi

Model	Number of cases	OR (95% Cl)	Pseudo R^2^	P value
MRP8/14 only	330	1.00 (0.99 to 1.01)	0.0000	0.839
MRP8/14 adjusted for pre-treatment CRP	330	1.00 (0.99 to 1.02)	0.0005	0.686

CDAI, clinical disease activity index; CRP, C reactive protein; TNFi, tumour necrosis factor inhibitor.

As well as investigating CDAI as an alternative outcome, we also assessed changes in DAS28, changes in CDAI and individual measures of disease activity after 3 months on treatment using linear regression. Using TNFi as a whole or extreme analysis using high/low concentrations of MRP8/14, we observed no significant associations with pre-treatment MRP8/14 (online supplemental table 10).

### MRP8/14 changes over time in ADA responders

**Figure 5:**
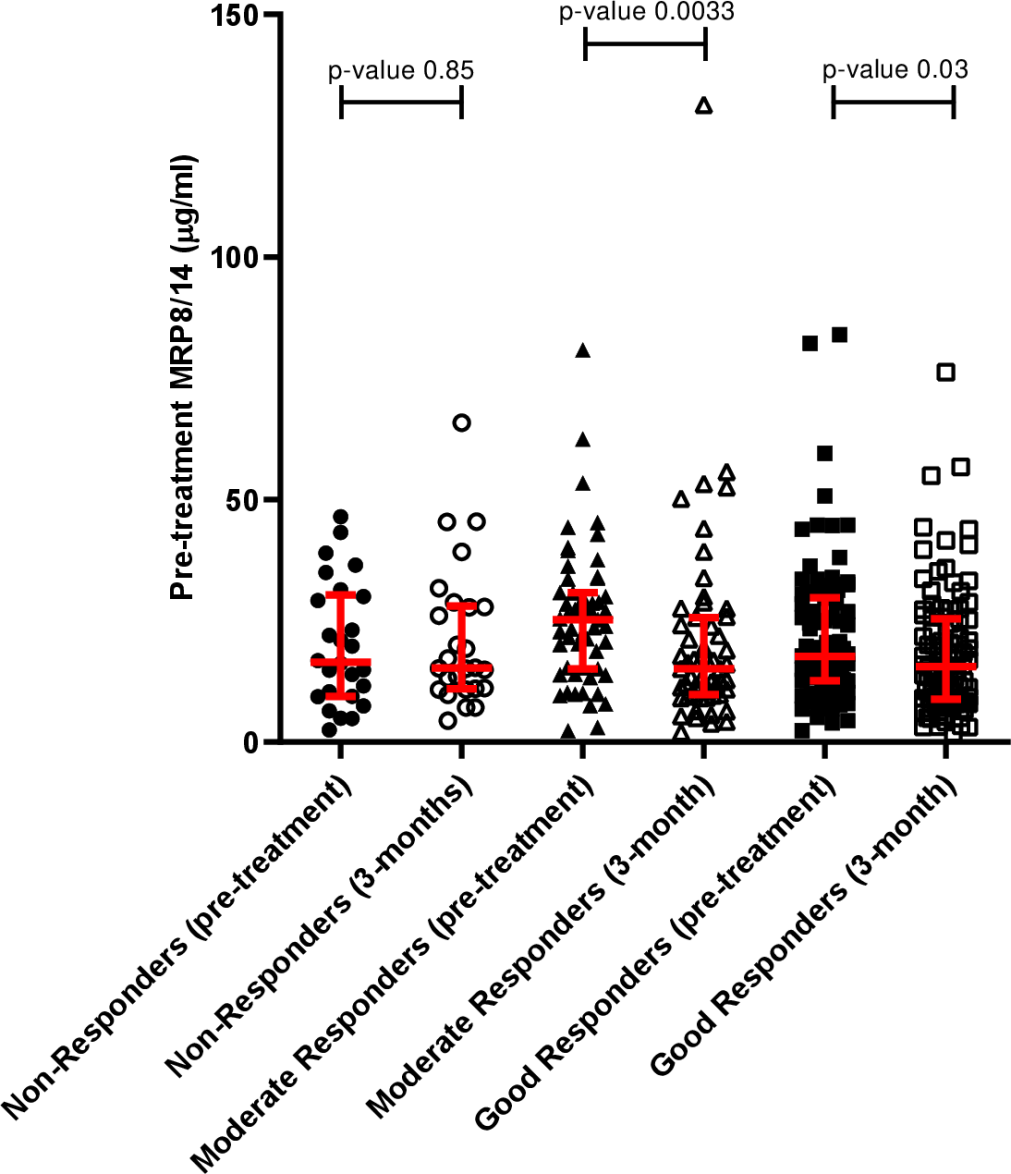
Changes in serum MRP8/14 after 3 months of treatment in adalimumab-treated patients. Concentrations are shown in EULAR non-responders, moderate responders and good responders, classified at 3-month follow-up. EULAR responses calculated using 3-component DAS28. Each dot represents a patient. The data is represented as median and interquartile range. Significance was determined using Mann-Whitney non-parametric test. EULAR, European League against Rheumatism.

Decreases in MRP8/14 have been shown to correlate with favourable response to treatment. Where paired pre-treatment and 3-month MRP8/14 were available for ADA-treated patients (n=179) in this study, we found that change in MRP8/14 over time correlated with response categories at 3 months (figure 5). In line with previous results, responders displayed a reduction in serum titres over time, while non-responders generally had constant or increased expression. For responders, pre-treatment MRP8/14 decreased by a median of 22.1% (−57.3% to −1.5%) and 21.5% (−51.2% to 28.4%) for moderate and good responders, respectively. Expression of MRP8/14 in non-responders to ADA treatment increased by a median of 7.8% (−25.2% to 99.9%) between pre-treatment and 3 months. Additionally, we were able to investigate change in MRP8/14 in a small subset of paired pre-treatment and 4-week samples (n=7; two non-responders and five good-responders). The average change after 4 weeks on treatment in non-responders (response assessed after 3 months on treatment) was +2.02 µg/mL, while good responders displayed a decrease of 2.68 µg/mL (online supplemental figure 8).

## Discussion

It is thought that the major source of MRP8/14 in arthritis is the synovial compartment with many studies reporting increased concentrations in inflamed synovial tissue and synovial fluid.^35^ In terms of a clinically applicable biomarker, it is not always possible to obtain synovial fluid, with serum representing a much more accessible option. Here, in the largest study conducted to date, we have shown correlations between pre-treatment serum MRP8/14 and measures of disease activity (DAS28-CRP, CRP and ESR), consistent with previous reports performed in serum. As a whole, we found that MRP8/14 was only associated with moderate treatment response. However, this was only observed in patients receiving ADA. This may be a power issue and now requires follow-up in larger sample numbers to determine whether the average difference observed is biologically meaningful. We also observed that pre-treatment MRP8/14 correlated with EULAR response, but only in those patients with high concentrations and this was irrespective of drug used. We were also able to show that CRP alone correlates more strongly with EULAR and adding MRP8/14 provided no additional information.

Strengths of the current study include, first, the large sample size tested in comparison to previously published studies, thereby reducing the chance of both false-negative and false-positive results. Second, we used a commercial CE-marked IVD ELISA kit to measure levels as used in previous reports. Finally, we explored different outcome measures of response and found that CRP was a better predictor of EULAR response compared with MRP8/14, when using DAS28-based outcome measures. Most studies of MRP8/14 reported to date used composite outcome measures that include acute phase proteins (CRP/ESR) to classify response to treatment (EULAR/ACR), but, due to the correlation between MRP8/14 and CRP/ESR, this could confound interpretation. A strength of the current study is that we also investigated use of the CDAI outcome measure, which does not include CRP/ESR. No additional improvement in model fit and therefore utility was gained by inclusion of both predictor proteins over CRP alone. Our findings support the notion that, beyond CRP, MRP8/14 provides no additional value in predicting response to TNFi treatment in RA, irrespective of the outcome measure used.

Interestingly, we observed that for both CRP and MRP8/14, the associations were only observed when used to predict 3C and 2C DAS28-CRP response categories, but not the 4C-derived model. We determined this was due to inclusion of the subjective patient VAS in the calculation of DAS28. We and others have reported that the VAS correlates with psychological measures,^36^ and it is perhaps not surprising that biological measures do not correlate well with composite outcome measures incorporating variables that correlate more strongly with psychological states.

We have shown that changes in MRP8/14 after 3 months on-treatment correlate with EULAR responses; however, this is not predictive of response, and ideally, an earlier time-point should have been explored as shown in previous studies after 4-week on-treatment.^16^ A limitation of the current study is that samples collected at 4 weeks’ post-treatment initiation were only available for a small subset of patients.

In the current study, we did not observe a correlation between MRP8/14 and SJC as has been reported in previous serum studies^10 16^; however, there are important differences in the way the SJC was measured between the current study and those previously reported. We counted in 28 joints, while previous studies included a more comprehensive 66/68 joint count. An additional study^23^ reported a correlation within 28 joints; however, this was only observed after adjusting for rheumatoid factor (RF) status and unfortunately we do not routinely collect RF to permit similar analyses. Others have shown that MRP8/14 is a superior marker of synovitis and disease activity (as measured by US) and have suggested its use over traditional acute phase reactants. The lack of association with SJC in this study may also be due in part to being unable to detect subtle/subclinical joint inflammation during the clinical examination of patients, whereas US is more able to detect subclinical inflammation. Whether this marker should replace CRP/ESR in composite scores, such as DAS28, requires further critical investigation, especially given the lack of association with CDAI responses shown here.

Compared with previous studies, we observed relatively high levels of MRP8/14. High MRP8/14 may result from various factors, including sample preparation (ie, delays in sample processing). Mylemans *et al* 37 observed in healthy controls that serum MRP8/14 increases significantly (60–80% increase) with centrifugation delays of 6 hours; however, this increase was less noticeable in patients with RA with already elevated levels. In the current study, we observed significant increases in MRP8/14 following >2 days delay to centrifugation, compared with samples processed within 8 hours. Use of serum samples could therefore be considered a limitation of the current study and testing in plasma samples (which is more robust against this pre-analytical vulnerability) should be considered for future studies. However, all samples were collected using the same study protocol, irrespective of future responder status, limiting the impact of this pre-analytical vulnerability on the interpretation of the results. Indeed, pre-analytical variability is not a desirable characteristic for clinically usable biomarkers and CRP, more robust to pre-analytical variability, should arguably be preferred.

Relatively high MRP8/14 values may also in part relate to the high levels of inflammation within the BRAGGSS cohort, where 48.3% of the cohort presented with marked/highly elevated levels of CRP. Further, there is also evidence to suggest that the BÜHLMANN assay reports higher MRP8/14 compared with other ELISAs.^19^ When investigating interassay variation, a previous report found that the BÜHLMANN ELISA yielded concentrations threefold to fourfold higher compared with their in-house ELISA; however, a strong correlation was observed (R^2^=0.90). Importantly, this difference did not materially alter their findings, just the thresholds used. In the current study, we observed a similarly strong correlation between the BÜHLMANN and BioLegend LEGEND MAX assays (R^2^=0.93) and the BÜHLMANN assay yielded concentrations three times higher. Use of the BÜHLMANN assay is therefore likely to contribute towards the high levels of MRP8/14 observed in the current study.

A limitation to the current study is that the BRAGGSS cohort was initially launched to investigate genetic predictors of treatment response. Due to differences between ethnically diverse populations and complexities in analysing multi-ethnic data, until recently, recruitment to BRAGGSS was limited to those of European descent. As a result, the availability of samples from patients of non-European ancestry is low (<5% of BRAGGSS), meaning that power considerations prevented analysis in those populations.

Unlike cytokines,^38^ MRP8/14 is highly stable, can be measured in serum without the need for cold storage, can be detected at relatively low levels and is already in clinical use in IBD.^39^ MRP8/14 could therefore represent a viable biomarker to enter routine clinical use in RA and, importantly, we found that time to processing was not a confounder, supporting the stability of the protein. However, CRP is similarly stable, is not subject to pre-analytical vulnerability and is cheaper to measure and correlates better with outcome.

Various studies in RA and JIA have also reported MRP8/14 as a potential marker for predicting disease flare/relapse following treatment termination.^40–42^ For example, Gerss *et al*,^43^ identified MRP8/14 as a way of identifying patients with JIA, in clinical remission but not subclinical/immunological remission, who are at increased risk of disease flare within 6 months of stopping treatment. Conversely, some studies have reported no associations between MRP8/14 and disease flare.^44 45^ A limitation of the current study is that we were not able to explore the association with flares due to lack of data.

In conclusion, while we have observed similar associations between pre-treatment MRP8/14 and measures of disease activity, associations between high MRP8/14 and response categories, and changes in MRP8/14 following successful treatment as reported previously, we found no evidence to suggest that MRP8/14 explains any additional variability in response to TNFi beyond the correlation with CRP, in patients with RA.

## Data Availability

Data are available upon request to the authors.
