# Supplementary material for: Pre-treatment calprotectin (MRP8/14) provides no added value to testing CRP alone in terms of predicting response to TNF inhibitors in rheumatoid arthritis in a post hoc analysis
Source: Ann Rheum Dis. 2023 Feb 21;82(5):611–20. doi: 10.1136/ard-2022-222519 (PMC10176427; doi:10.1136/ard-2022-222519)
Supplement: Supplementary data [file ard-2022-222519supp001.pdf]

Supplementary Material

	4C (n=434)			3C (n=470)			2C (n=470)		
	TNFi	ADA only	ETN only	TNFi	ADA only	ETN only	TNFi	ADA only	ETN only
**EULAR response at 3-months, n (%):**									
Non-responder	79 (18.2)	27 (15.2)	52 (20.3)	91 (19)	33 (17)	58 (21)	88 (18.7)	35 (17.9)	53 (19.3)
Moderate responder	181 (41.7)	67 (37.6)	114 (44.5)	172 (37)	63 (32)	109 (40)	99 (21.1)	33 (16.8)	66 (24.1)
Good responder	174 (40.1)	84 (47.2)	90 (35.2)	207 (44)	100 (51)	107 (39)	283 (60.2)	128 (65.3)	155 (56.6)

Suppl Table 1: Breakdown of EULAR responses after 3-months on treatment calculated using 4-component, 3-component and 2-compnent DAS28 scores.

	Responses categories calculated using								
	4-component DAS28			3-component DAS28			2-component DAS28		
**Model**	**Number of cases**	**Odds Ratio (95% CI)**	**P-value**	**Number of cases**	**Odds Ratio (95% CI)**	**P-value**	**Number of cases**	**Odds Ratio (95% CI)**	**P-value**
**Pre-treatment MRP8/14**	434	1.00 (0.98-1.02)	0.96	470	1.00 (0.99-1.02)	0.38	470	1.00 (0.99-1.02)	0.36
**Pre-treatment CRP**	434	1.00 (0.99-1.02)	0.475	470	1.01 (1.00-1.03)	0.034	470	1.01 (1.00-1.03)	0.044

Suppl Table 2: Using all available cases, comparison of logistic regression models for response to TNFi, comparing continuous MRP8/14 or continuous CRP to grouped responders (moderate and good) and non-responders at 3-month follow-up. Response categories were calculated using either 4-components, 3-components or 2-components.

	Responses categories calculated using								
	4-component DAS28			3-component DAS28			2-component DAS28		
**Model**	**Number of cases**	**Odds Ratio (95% CI)**	**P-value**	**Number of cases**	**Odds Ratio (95% CI)**	**P-value**	**Number of cases**	**Odds Ratio (95% CI)**	**P-value**
Pre-treatment CRP limiting to n=434 in which can calculate 4C DAS28	-	-	-	434	1.01 (1.00-1.03)	0.062	434	1.01 (1.00-1.03)	0.05

Suppl Table 3: Logistic regression for response to TNFi, comparing continuous CRP to grouped responders (moderate and good) and non-responders at 3-month follow-up, limiting to only those patients with 4-component DAS28 responses.

	Responses categories calculated using:											
	4-component DAS28				3-component DAS28				2-component DAS28			
**Model**	**Cases**	**Odds Ratio (95% CI)**	**Model Fit (Pseudo R2)**	**p-value**	**Cases**	**Odds Ratio (95% CI)**	**Model Fit (Pseudo R2)**	**p-value**	**Cases**	**Odds Ratio (95% CI)**	**Model Fit (Pseudo R2)**	**p-value**
**Low vs. high MRP8/14 limiting to n=218 in which can calculate 4C=DAS28**	-	-	-	-	218	1.97 (1.02-3.79)	0.018	0.044	218	1.88 (0.97-3.63)	0.016	0.062
**Low vs. high CRP limiting to n=215 in which can calculate 4C=DAS28**	-	-	-	-	215	3.82 (1.76-8.29)	0.063	0.001	215	3.82 (1.76-8.29)	0.063	0.001

Suppl Table 4: Logistic regression models for response to TNFi, comparing grouped responders (moderate and good) and non-responders at 3-month follow-up, dichotomised using high (75th) and low (25th) concentrations of MRP8/14 or CRP and limiting to only those patients with 4-component DAS28 responses.

	Response categories calculated using:								
	4-component DAS28			3-component DAS28			2-component DAS28		
**Model**	**Cases**	**Odds Ratio (95% CI)**	**p-value**	**Cases**	**Odds Ratio (95% CI)**	**p-value**	**Cases**	**Odds Ratio (95% CI)**	**p-value**
**Low vs. high MRP8/14 (µg/ml)**	218	1.22 (0.62-2.38)	0.56	241	1.92 (1.04-3.54)	0.037	241	2.03 (1.09-3.78)	0.025
**Low vs. high adjusted for delay (days)**	95			101			101		
MRP8/14		1.21 (0.38-3.92)	0.75		1.74 (0.57-5.29)	0.33		1.73 (0.58-5.14)	0.32
Processing delay		1.10 (0.71-1.70)	0.66		1.18 (0.77-1.79)	0.45		1.03 (0.69-1.53)	0.89

Suppl Table 5: Logistic regression models for response to TNFi, comparing grouped responders (moderate and good) and non-responders at 3-month follow-up, dichotomised based using high (75th) and low (25th) concentrations of MRP8/14 and adjusted for delays in sample processing (days from blood sampling to centrifugation). Response categories were calculated using either 4C-, 3C- or 2C-DAS28-CRP.

Model	Drug	Number of cases	Odds Ratio (95% CI)	Model Fit (Pseudo R2)	p-value
**Low vs. high MRP8/14 (µg/ml)**	ADA	93	1.11 (0.39-3.17)	0.0004	0.85
**Low vs. high MRP8/14 adjusted for pre-treatment CRP**	ADA	93	1.56 (0.45-5.37)	0.015	0.48

**Low vs. high CRP (mg/L)**	ADA	94	1.26 (0.41-3.86)	0.0019	0.69
**Low vs. high CRP adjusted for pre-treatment MRP8/14**	ADA	94	1.26 (0.36-4.4)	0.0019	0.72
**Low vs. high MRP8/14 (μg/ml)**	ETN	125	1.33 (0.55-3.2)	0.0032	0.53
**Low vs. high MRP8/14 adjusted for pre-treatment CRP**	ETN	125	0.95 (0.37-2.4)	0.030	0.92
**Low vs. high CRP (mg/L)**	ETN	121	2.02 (0.76-5.38)	0.019	0.16
**Low vs. high CRP adjusted for pre-treatment MRP8/14**	ETN	121	2.32 (0.79-6.8)	0.022	0.12

Suppl Table 6: Comparison of different logistic regression models comparing grouped responders (moderate and good) and non-responders at 3-month follow-up stratified by drug type. Response categories were calculated using 4C-DAS28-CRP scores.

Model	Drug	Number of cases	Odds Ratio (95% Cl)	Model Fit (Pseudo R2)	p-value
**Low vs. high MRP8/14 adjusted for pre-treatment CRP**	ADA	106	1.72 (0.64-4.6)	0.011	0.28
	ADA	106	2.00 (0.65-6.1)	0.014	0.22
**Low vs. high CRP (mg/L)**					
**Low vs. high CRP adjusted for pre-treatment MRP8/14**	ADA	106	2.86 (0.96-8.58)	0.038	0.060
	ADA	106	2.76 (0.84-9.11)	0.038	0.095
**Low vs. high MRP8/14 (μg/ml)**					
**Low vs. high MRP8/14 adjusted for pre-treatment CRP**	ETN	135	2.20 (0.98-4.92)	0.025	0.055
	ETN	135	1.42 (0.60-3.35)	0.071	0.425
**Low vs. high CRP (mg/L)**					
**Low vs. high CRP adjusted for pre-treatment MRP8/14**	ETN	128	4.8 (1.76-13.1)	0.087	0.002
**Low vs. high MRP8/14 adjusted for pre-treatment CRP**	ETN	128	5.69 (1.88-17.2)	0.0917	0.002

Suppl Table 7: Comparison of different logistic regression models comparing grouped responders (moderate and good) and non-responders at 3-month follow-up stratified by drug type. Responses categories were calculated using 3C-DAS28-CRP scores.

Model	Drug	Number of cases	Odds Ratio (95% Cl)	Model Fit (Pseudo R2)	p-value
**Low vs. high MRP8/14 (µg/ml)**	ADA	106	2.00 (0.76-5.3)	0.018	0.16
**Low vs. high MRP8/14 adjusted for pre-treatment CRP**	ADA	106	2.00 (0.68-5.94)	0.018	0.21
**Low vs. high CRP (mg/L)**	ADA	106	4.72 (1.47-15.1)	0.077	0.009
**Low vs. high CRP adjusted for pre-treatment MRP8/14**	ADA	106	4.57 (1.3-16.1)	0.078	0.018
**Low vs. high MRP8/14 (µg/ml)**	ETN	135	2.03 (0.89-4.66)	0.020	0.095
**Low vs. high MRP8/14 adjusted for pre-treatment CRP**	ETN	135	1.40 (0.58-3.41)	0.051	0.45
**Low vs. high CRP (mg/L)**	ETN	128	2.95 (1.16-7.52)	0.044	0.023
**Low vs. high CRP adjusted for pre-treatment MRP8/14**	ETN	128	3.23 (1.16-8.99)	0.046	0.025

Suppl Table 8: Comparison of different logistic regression models comparing grouped responders (moderate and good) and non-responders at 3-month follow-up stratified by drug type. Responses categories were calculated using 2C-DAS28-CRP scores.

Model	Drug	Number of cases	Odds Ratio (95% Cl)	Model Fit (Pseudo R2)	p-value	LR test chi2 (p-value)
**MRP8/14 only**	ADA	140	1.00 (0.98-1.02)	0.0000	0.947	
**MRP8/14 adjusted for pre-treatment CRP**	ADA	140	1.00 (0.98-1.02)	0.0009	0.818	0.33 (0.57)
**MRP8/14 only**	ETN	190	1.00 (0.99-1.02)	0.0002	0.728	
**MRP8/14 adjusted for pre-treatment CRP**	ETN	190	1.00 (0.99-1.02)	0.0006	0.636	0.18 (0.67)

Suppl Table 9: Results from ordered logistic regression models to determine the association between pre-treatment MRP8/14 and CDAI responses by drug type. ADA = adalimumab; ETN = etanercept; CI = confidence interval; LR test = likelihood ratio test to determine improvement in model fit.

Model	All TNFi cases			High/Low MRP8/14		
	B Coef (95% CI)	Std Err	p-value	B Coef (95% CI)	Std Err	p-value
**Change in 4C DAS28**	-0.18 (-0.38-0.28)	0.10	0.091	-0.20 (-0.41-0.018)	0.11	0.072
**Change in 3C DAS28**	-0.16 (-0.33-0.022)	0.091	0.086	-0.18 (-0.37-0.015)	0.097	0.071
**Change in 2C DAS28**	-0.15 (-0.36-0.060)	0.11	0.16	-0.16 (-0.39-0.068)	0.12	0.17
**Change in CDAI**	-0.39 (-2.90-2.12)	1.28	0.76	-0.63 (-3.31-2.06)	1.36	0.65
**Change in TJC**	-0.22 (-1.26-0.81)	0.53	0.67	-0.34 (-1.44-0.76)	0.56	0.55
**Change in SJC**	-0.0037 (-0.82-0.82)	0.42	0.99	-0.026 (-0.94-0.89)	0.46	0.96
**Change in Physicians Global**	0.64 (-3.55-4.84)	2.13	0.76	0.36 (-3.99-4.70)	2.20	0.87
**Change in Patient Global**	-1.62 (-5.74-2.50)	2.10	0.44	-1.66 (-5.85-2.52)	2.12	0.43

Suppl Table 10: Linear regression of changes in DAS28, change in CDAI and change in individual measures of disease activity after 3-months of TNFi treatment. Analysis stratified by all available cases (n=470) and extreme (75th/25th) MRP8/14.

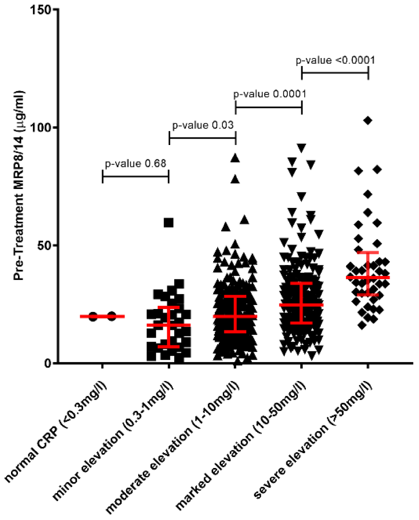

Suppl Figure 1: Pre-treatment MRP8/14 stratified by pre-treatment levels of CRP.

Each dot represents a patient. The data is represented as median and inter-quartile range. Significance was determined using Mann-Whitney non-parametric test.

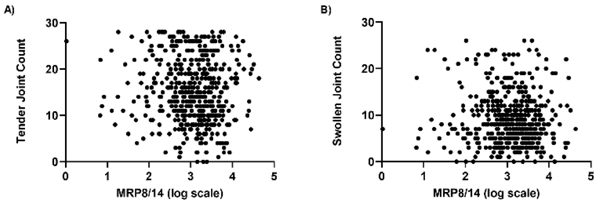

Suppl Figure 2: Scatterplots showing the correlation between pre-treatment serum levels of MRP8/14 and parameters of baseline disease activity: A) tender joint count at 28-joints, B) swollen joint count at 28-joints.

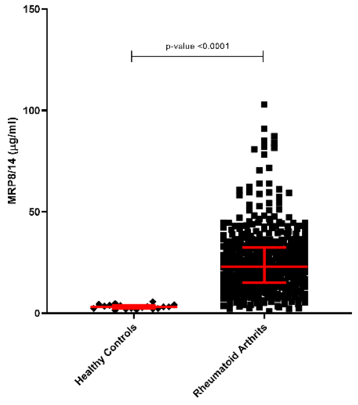

Suppl Figure 3: MRP8/14 measured in healthy controls and all pre-treatment RA samples

Each dot represents a patient. The data is represented as median and inter-quartile range. Significance was determined using Mann-Whitney non-parametric test.

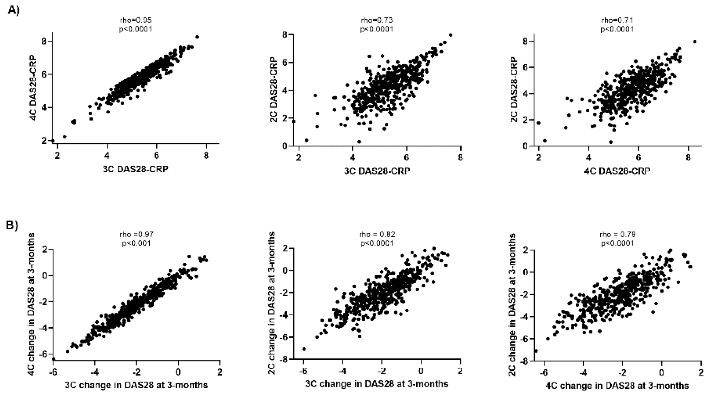

Suppl Figure 4: Scatterplots showing the correlation between A) pre-treatment DAS28-CRP scores calculated using 4, 3 and 2-components and B) change in DAS28-CRP, calculated using 4, 3 and 2-components, after 3 months of TNFi treatment. DAS28: disease activity scores in 28-joints.

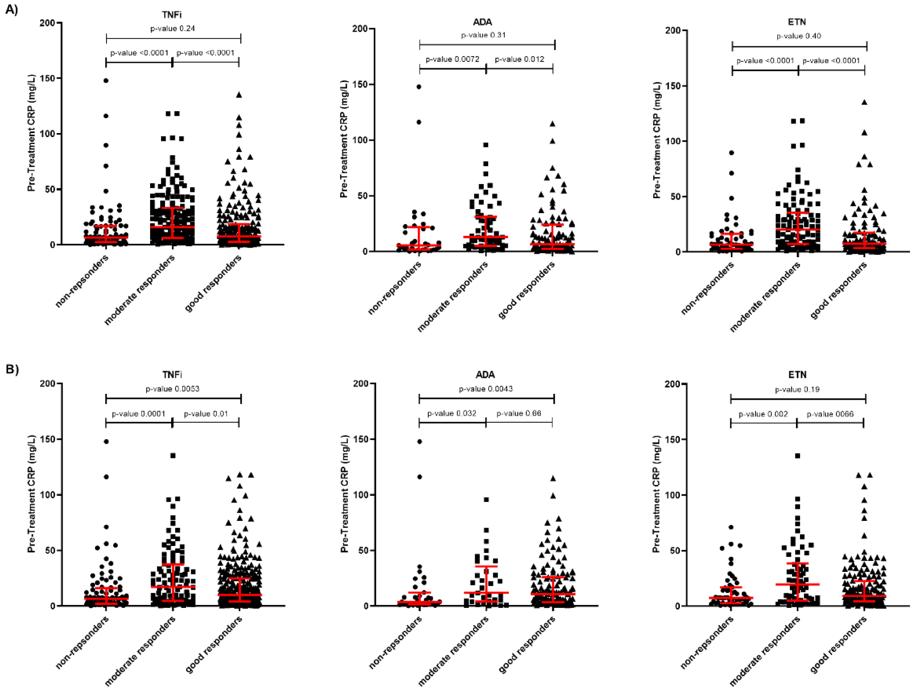

Suppl Figure 5 A) Pre-treatment CRP by A) 3C EULAR responses B) 2C EULAR responses
Pre-treatment CRP against EULAR non-responders, moderate responders and good responders, classified at 3-month follow-up to TNF inhibitors as a whole (left-hand plots), adalimumab (ADA) only (middle plots) and etanercept (ETN) only (right-hand plots). Each dot represents a patient. The data is represented as median and inter-quartile range. Significance was determined using Mann-Whitney non-parametric test.

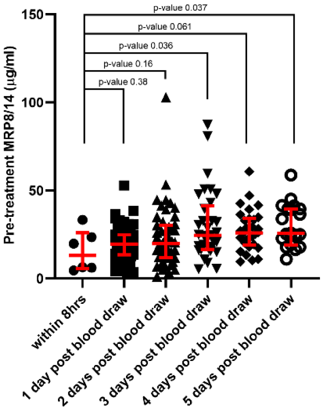

Suppl Figure 6: The relationship between delays to centrifugation and levels of MRP8/14

Each dot represents a patient. The data is represented as median and inter-quartile range. Significance was determined using multinomial logistic regression. Samples processed the same day as blood sampling (within 8hrs) was used as a baseline comparison group.

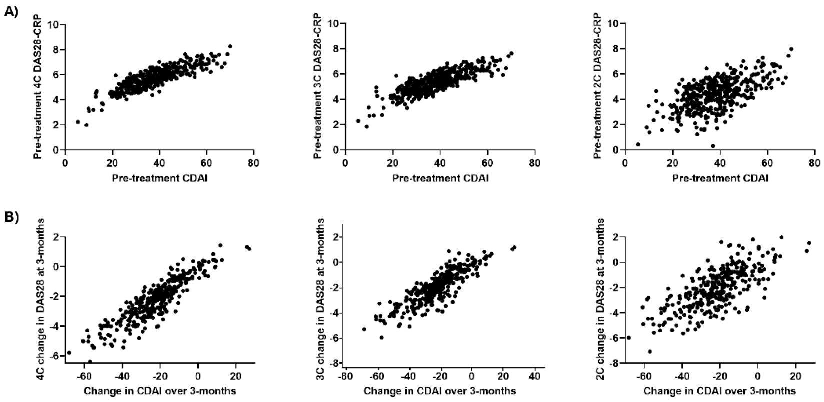

Suppl figure 7: Scatterplots showing the correlation between A) pre-treatment CDAI and pre-treatment DAS28-CRP and B) change in CDAI and change in DAS28-CRP after 3 months of TNFi treatment. DAS28-CRP was calculated using 4-components (left plots), 3-components (middle plots) and 2-components (right plots). DAS28: disease activity scores in 28-joints; CDAI: clinical disease activity index.

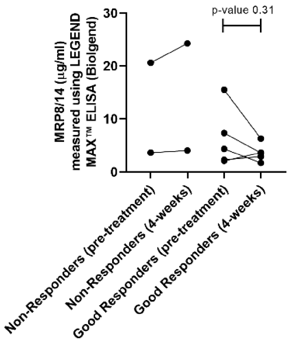

Suppl Figure 8: Changes in serum MRP8/14 after 4-weeks on treatment in TNFi treated patients. MRP8/14 was determined using the LEGEND MAX™ Human MRP8/14 (Calprotectin) ELISA kit (Biolegend). Concentrations are shown in EULAR non-responders and good responders, classified at 3-month follow-up using 3-component DAS28-CRP. Significance was determined using a paired-Wilcoxen non-parametric test (for the good responder group only).
